# Construction of a Non‐Invasive Predictive Model Based on PIVKA‐II Combined With MRI Imaging Features for Evaluating Microvascular Invasion in Hepatocellular Carcinoma

**DOI:** 10.1002/kjm2.70203

**Published:** 2026-03-16

**Authors:** Di Gao, Rui‐Qi Jin, Hong‐Wei Wang

**Affiliations:** ^1^ Department of Radiology Harbin Medical University Cancer Hospital Harbin Heilongjiang China

**Keywords:** hepatocellular carcinoma, magnetic resonance imaging, microvascular invasion, non‐invasive prediction model, prothrombin induced by vitamin K absence or antagonist‐II (PIVKA‐II)

## Abstract

This study developed a non‐invasive model using PIVKA‐II and MRI features to predict microvascular invasion in hepatocellular carcinoma, providing a reliable tool for early risk assessment and personalized treatment planning. The study included 98 patients with pathologically confirmed HCC (Child‐Pugh A, BCLC stage A), comprising 43 MVI‐positive and 55 MVI‐negative cases. Baseline clinical characteristics and MRI features were collected. Univariate analysis identified candidate variables associated with MVI, which were subsequently included in multivariate logistic regression analysis to identify independent influencing factors. A nomogram prediction model was developed based on the independent factors. The model's diagnostic performance, calibration, and clinical applicability were evaluated. The diagnostic value of PIVKA‐II alone for MVI was analyzed. The MVI‐positive group showed significantly higher alpha‐fetoprotein ≥ 400 ng/mL, PIVKA‐II levels, and aspartate aminotransferase levels. ROC curve analysis showed that PIVKA‐II alone had an AUC of 0.682 for diagnosing MVI, with a maximum Youden's index of 33.57, a cut‐off value of 741.5 mAU/mL, 37.21% sensitivity, 96.36% specificity. Tumor diameter, peritumoral abnormal enhancement, intratumoral arteries, and mean tumor ADC value showed significant differences between the two groups. Elevated PIVKA‐II, intratumoral arteries, and mean tumor ADC value were independent influencing factors for MVI in HCC. A nomogram incorporating these factors achieved an AUC of 0.81, outperforming PIVKA‐II alone. The model demonstrated good calibration and clinical utility. The non‐invasive predictive nomogram constructed by combining PIVKA‐II with MRI features demonstrates good predictive value and clinical applicability for assessing MVI in HCC patients. [Correction added on 25 March 2026, after first online publication: In the sentence “A nomogram incorporating these factors achieved.......” the value “0.85” is changed to “0.81” in this version.]

## 1 Introduction

Hepatocellular carcinoma (HCC) is a malignancy with high global incidence and mortality rates, posing persistent clinical challenges related to tumor recurrence, metastasis, and prognostic evaluation [1, 2]. Although early radical resection remains an effective treatment, the 5‐year recurrence‐free survival rate remains unsatisfactory, ranging only from 26% to 40% [3]. Among various pathological factors influencing postoperative recurrence risk and long‐term survival, microvascular invasion (MVI) is widely recognized as a key prognostic indicator [4, 5]. The occurrence of MVI is a complex, multifactorial process whose precise mechanisms are not fully understood. Some studies suggest it may be related to tumor cells secreting growth and chemotactic factors, which induce the proliferation and migration of microvascular endothelial cells to form new vessels, thereby promoting tumor cell invasion and metastasis [6, 7, 8]. Furthermore, MVI has been associated with tumor cell immune evasion via the microvascular network [9, 10]. Many studies indicate that MVI is a significant risk factor for tumor recurrence and poor prognosis in HCC patients [11]. It is estimated that approximately 15% to 57% of HCC patients have MVI. The 5‐year disease‐free survival (DFS) and overall survival (OS) rates for HCC patients with MVI are 7.5%–48% and 38.4%–66%, respectively, which are significantly lower than those for patients without MVI [12]. Accurate preoperative or pre‐interventional identification of MVI status can provide essential guidance for selecting surgical margins, formulating adjuvant therapy strategies, and optimizing follow‐up protocols, thereby providing indispensable clinical value for improving patient outcomes [13, 14, 15].

Currently, postoperative histopathological examination remains the gold standard for MVI detection. This method is limited to evaluating postoperative paraffin‐embedded tissue sections and thus suffers from inherent delay. Moreover, as MVI is often distributed in peritumoral liver tissue, multiple sampling around the tumor is necessary to ensure detection accuracy [16]. This requirement further limits its application in preoperative assessment. Therefore, there is a clinical urgency to establish an efficient, non‐invasive preoperative evaluation protocol to achieve accurate prediction of MVI in HCC.

To overcome the limitations of postoperative pathological assessment, researchers worldwide have extensively explored non‐invasive preoperative predictive markers for MVI, and the predictive value of a series of serum biomarkers has been gradually investigated and validated. The Fibrosis‐4 (FIB‐4) index, a non‐invasive marker calculated from platelet count, aspartate aminotransferase (AST), alanine aminotransferase (ALT), and age for assessing liver fibrosis, has been shown in some studies to correlate with the occurrence of MVI in HCC, where a high FIB‐4 index may indicate an increased risk of MVI [17]. The Albumin‐Bilirubin (ALBI) grade is a simple method for assessing liver function by calculating a score based on serum albumin and bilirubin levels. Research has found an association between the ALBI grade and the prognosis as well as MVI status in HCC patients, with a lower incidence of MVI potentially observed in patients with lower ALBI scores [18]. Furthermore, Protein Induced by Vitamin K Absence or Antagonist‐II (PIVKA‐II), a HCC‐specific serum biomarker that has attracted considerable interest in recent years, shows elevated levels closely associated with tumor differentiation and invasiveness [19]. Previous studies have suggested its potential predictive value for MVI [20, 21]. However, when used individually, the predictive performance of these serum markers for MVI remains suboptimal and insufficient to meet clinical demands for accuracy in MVI assessment. Meanwhile, magnetic resonance imaging (MRI), with its high soft‐tissue resolution and multi‐parametric imaging capabilities, enables detailed visualization of tumor characteristics such as size, morphology, capsule integrity, enhancement patterns, and apparent diffusion coefficient (ADC) values on diffusion‐weighted imaging (DWI). These imaging features may not only reflect tumor biological behavior but also correlate with the presence of MVI [22, 23]. Recent studies have further reported associations between MRI features—including tumor capsule, peritumoral enhancement, and tumor margin—and MVI [24]. In research on non‐invasive assessment methods, the combined application of serum tumor markers and imaging technology has gradually become a focal point.

Although PIVKA‐II and MRI features individually demonstrate certain predictive potential for MVI, the diagnostic performance of either approach alone remains suboptimal and insufficient to meet clinical requirements for accuracy. Therefore, integrating serum biomarkers with imaging characteristics to develop a multidimensional non‐invasive predictive model may combine their advantages and improve the accuracy and reliability of MVI prediction. Based on this rationale, this study aims to explore the feasibility of constructing a non‐invasive predictive model by combining PIVKA‐II and MRI features, and to evaluate its clinical value in assessing MVI in HCC patients. The model is anticipated to provide a reliable non‐invasive tool for early risk assessment of MVI and support individualized treatment planning, ultimately contributing to improved prognosis and optimized clinical decision‐making for HCC patients.

## 2 Materials and Methods

### 2.1 Study Subjects

A retrospective analysis was conducted on 117 patients admitted to our hospital between March 2024 and June 2025.

Inclusion criteria: (1) Patients diagnosed with HCC according to the Guidelines for the Diagnosis and Treatment of Primary Liver Cancer (2022 Edition), aged ≥ 18 years; (2) patients classified as Child‐Pugh Class A or B; (3) patients who underwent surgical resection with postoperative pathological confirmation of HCC and definitive MVI status (MVI‐positive or MVI‐negative), with MVI defined as the presence of tumor cell clusters within microvessels (including central veins, portal vein branches, and capsular venules), tumor cells identified within the vascular endothelium or smooth muscle layer, or tumor cells detected in association with fibrin or red blood cell aggregates in vascular lumina; (4) patients with no evidence of distant metastasis confirmed by preoperative imaging and clinical examination; (5) patients with solitary tumors; (6) patients with no history of hepatic resection, liver transplantation, chemotherapy, radiotherapy, or immunosuppressive therapy for HCC; (7) Patients with complete clinical, pathological, serological, and radiological data, including contrast‐enhanced abdominal MRI performed at our institution within 2 weeks prior to surgery.

Exclusion criteria: (1) Patients with non‐primary liver cancer or concurrent other malignancies; (2) patients with postoperative pathological findings indicating intrahepatic cholangiocarcinoma or combined hepatocellular‐cholangiocarcinoma, or without MVI assessment; (3) patients with a history of prior antitumor therapy, including transarterial chemoembolization, percutaneous radiofrequency ablation, liver transplantation, targeted therapy, or immunotherapy; (4) patients with radiological or pathological evidence of portal vein invasion, intrahepatic metastasis, hilar lymph node involvement, or distant metastasis; (5) patients lacking complete clinical, laboratory, or follow‐up data; (6) patients with incomplete or poor‐quality imaging studies.

### 2.2 Laboratory Indicator Testing

Within 1 week before surgery, 5 mL of venous blood was collected from the antecubital vein of each patient. The blood samples were centrifuged at 3800 rpm (r/min) with a centrifugal radius of 13.5 cm for 12 min to obtain the supernatant. PIVKA‐II levels were measured using a fully automated chemiluminescence immunoassay system (Fujirebio LUMIPULSE G1200) with matched reagent kits. Alpha‐fetoprotein (AFP) was detected using a Roche Cobas E602 electrochemiluminescence immunoanalyzer and corresponding reagents. ALT, AST, albumin (ALB), and total bilirubin (TBIL) were analyzed using a Siemens fully automated biochemical analyzer and compatible assay kits.

### 2.3 MRI Examination Protocol

MRI scanning was performed using a GE Discovery MR750 3.0 T system (General Electric, USA) with an 8‐channel phased‐array surface coil for signal reception. All patients provided written informed consent for contrast agent administration. After contraindications were excluded, patients were instructed to wear cotton clothing, remove metallic objects, and remain still during the examination.

Patients were placed in a supine position, head‐first, and asked to breathe gently to minimize motion artifacts. Both arms were raised to avoid inclusion in the scan field. The imaging coverage extended from the diaphragmatic dome to the inferior edge of the liver.

The following sequences and parameters were used: T1‐weighted imaging (T1WI): Liver Acceleration Volume Acquisition sequence; repetition time (TR) 3.7 ms, echo time (TE) 1.7 ms, matrix 260 × 150, field of view (FOV) 380 mm × 304 mm, slice thickness 5 mm. T2‐weighted imaging (T2WI): TR 2609 ms, TE 82.2 ms, matrix 320 × 320, FOV 360 mm × 360 mm, slice thickness 7 mm. DWI: TR 7059 ms, TE 78.4 ms, matrix 160 × 192, FOV 360 mm × 288 mm, slice thickness 7 mm; *b* values: 0, 800, 1000, and 1200 s/mm^2^.

After conventional sequences, gadoxetic acid disodium (Bayer Healthcare, Germany) was injected intravenously at a dose of 0.1 mmol/kg body weight using a power injector at a rate of 2–3 mL/s, followed by a 20 mL saline flush at the same rate. Axial dynamic contrast‐enhanced phases were acquired at 20–30 s (arterial phase), 60–90 s (portal venous phase), 3–5 min (transitional phase), and 20 min (hepatobiliary phase) using the same parameters as pre‐contrast T1WI. All examinations were performed by the same experienced radiologist.

### 2.4 Image Analysis

All imaging data of the enrolled patients were transferred to a Picture Archiving and Communication System. Two radiologists (with 8 and 10 years of experience, respectively), blinded to clinical information, independently evaluated and recorded the imaging features. Any discrepancies in assessment were resolved through consensus discussion. The analyzed indicators included:

Maximum tumor diameter: Measured on T2WI or on the portal venous/delayed phase of contrast‐enhanced scans, with the tumor capsule included.

Tumor signal intensity: Recorded as hypointense or isointense on T1WI, hyperintense or hypo‐/isointense on T2WI, and hyperintense or isointense on DWI, all relative to the surrounding normal liver parenchyma.

DWI/T2WI mismatch: Defined as a tumor diameter on DWI larger than that on T2WI at the slice showing the maximum tumor diameter, where the signal intensity in the mismatched area was lower than that of the main tumor but higher than the liver parenchyma on DWI.

Tumor morphology and margin: Evaluated on T2WI, late arterial phase, or portal venous phase images. Morphology was categorized as regular or irregular. Regular morphology referred to nodular tumors with a round or oval shape, smooth edges, and clear boundaries. Irregular morphology was defined by any of the following: lobulated or irregular shape with clear boundaries; oval shape with one or more localized nodular protrusions at the margin; confluent multinodular lesions where each nodule had clear boundaries; or infiltrative growth without a distinct boundary.

Peritumoral abnormal enhancement: Defined as an irregular enhancing area in the liver parenchyma around the tumor during the arterial phase, which became isointense to the background liver parenchyma in the delayed phase.

Intratumoral arteries: Defined as continuous or discontinuous, dilated, tortuous and disorganized small vascular shadows within the tumor during the arterial and/or portal venous phase.

Intratumoral mosaic architecture: Defined as the presence of a cluster of randomly distributed intratumoral nodules or septa within the tumor, which vary in shape, size, signal intensity, and degree of enhancement.

Tumor capsule: Assessed on the delayed phase of contrast‐enhanced scans. A capsule was considered intact if a smooth, uniform, and complete rim‐like enhancement was present at the tumor margin. It was considered non‐intact if there was one or more localized defects in the enhancing rim or if no distinct capsule was visualized.

Tumor exophytic growth: Determined by whether the tumor protruded beyond the liver contour.

Tumor enhancement pattern: Categorized as typical or atypical. Typical enhancement was defined as non‐rim arterial phase hyperenhancement with non‐peripheral washout on the portal venous or delayed phases. Atypical enhancement referred to rim, halo, or heterogeneous enhancement during dynamic contrast‐enhanced scans, with or without washout on the portal venous or delayed phases.

Mean tumor ADC value: The mean ADC value of the tumor's solid component was measured on the ADC map (*b* value = 800 s/mm^2^). Regions of interest (20–100 mm^2^) were placed to avoid peritumoral tissue, hemorrhage, necrosis, or cystic areas within the tumor. Measurements were taken at three different locations, and the results were averaged.

### 2.5 Statistical Analysis

IBM SPSS Statistics 22.0 was used for data processing. Categorical variables were described using numbers and percentages and compared between groups using the *χ* ^*2*^ test or Fisher's exact test. Continuous variables were first tested for normality using the Shapiro–Wilk test. Normally distributed data were expressed as mean ± standard deviation (*x¯* ± s) and compared using the independent samples *t*‐test; non‐normally distributed data were expressed as median (interquartile range) [M (P25, P75)] and compared using the Mann–Whitney *U* test. Diagnostic performance was evaluated by receiver operating characteristic (ROC) curve analysis, from which the area under the curve (AUC), cut‐off value, sensitivity, and specificity were determined. Univariate and multivariate logistic regression analyses were performed to identify influencing factors of MVI in HCC. A nomogram for predicting MVI was developed using R software version 3.6.1. The predictive performance of the nomogram was assessed using the Hosmer–Lemeshow goodness‐of‐fit test, calibration curve, and ROC analysis. Clinical utility was evaluated using decision curve analysis (DCA). A *p* value < 0.05 was considered statistically significant.

## 3 Results

### 3.1 Baseline Characteristics

The study initially enrolled 117 individuals with pathologically verified HCC. Following the exclusion of 12 subjects due to incomplete baseline information, 5 with undetermined MVI status, and 2 presenting with multifocal lesions, a total of 98 patients were retained for analysis (the inclusion process is shown in Figure 1). This cohort comprised 43 cases (43.88%) classified as MVI‐positive and 55 cases (56.12%) as MVI‐negative. All participants were graded as Child‐Pugh class A and Barcelona Clinic Liver Cancer (BCLC) Stage A. Comprehensive baseline clinical profiles for both groups are detailed in Table 1 and Figure 2. Evaluation of demographic and clinical parameters indicated that age and sex were not significantly different between the MVI‐negative and MVI‐positive groups (*p* > 0.05), implying that these factors may not substantially influence MVI occurrence. Furthermore, the distribution of HCC etiology (including hepatitis B, hepatitis C infection, and other causes), antiviral treatment status, and the presence of cirrhosis showed no statistically significant differences between the two groups (*p* > 0.05). Likewise, ALB and TBIL—biomarkers commonly associated with hepatic synthesis and metabolic capacity—showed comparable levels across groups (*p* > 0.05). Although ALT values were marginally elevated within the MVI‐positive cohort, this deviation did not attain statistical significance, indicating a need for further investigation into its potential relevance. Markedly divergent results were observed for AFP, PIVKA‐II, and AST. A significantly greater proportion of subjects in the MVI‐positive group exhibited AFP values ≥ 400 ng/mL, supporting a plausible correlation between elevated AFP and enhanced invasive potential facilitating MVI. Similarly, PIVKA‐II concentrations were substantially higher among MVI‐positive patients, implicating associated coagulation abnormalities as a concomitant feature of MVI. Increased AST levels within this group also suggested more pronounced hepatocellular injury, potentially fostering intravasation of malignant cells. ROC analysis was employed to assess the discriminative capacity of PIVKA‐II in detecting MVI (Figure 3). The AUC was computed as 0.682 (95% CI: 0.572–0.791). A maximum Youden's index of 33.57 corresponded to an optimal cutoff value of 741.5 mAU/mL, yielding a sensitivity of 37.21% and specificity of 96.36%. These outcomes underscore the limited efficacy of utilizing PIVKA‐II in isolation for MVI prediction in HCC patients.

**FIGURE 1:**
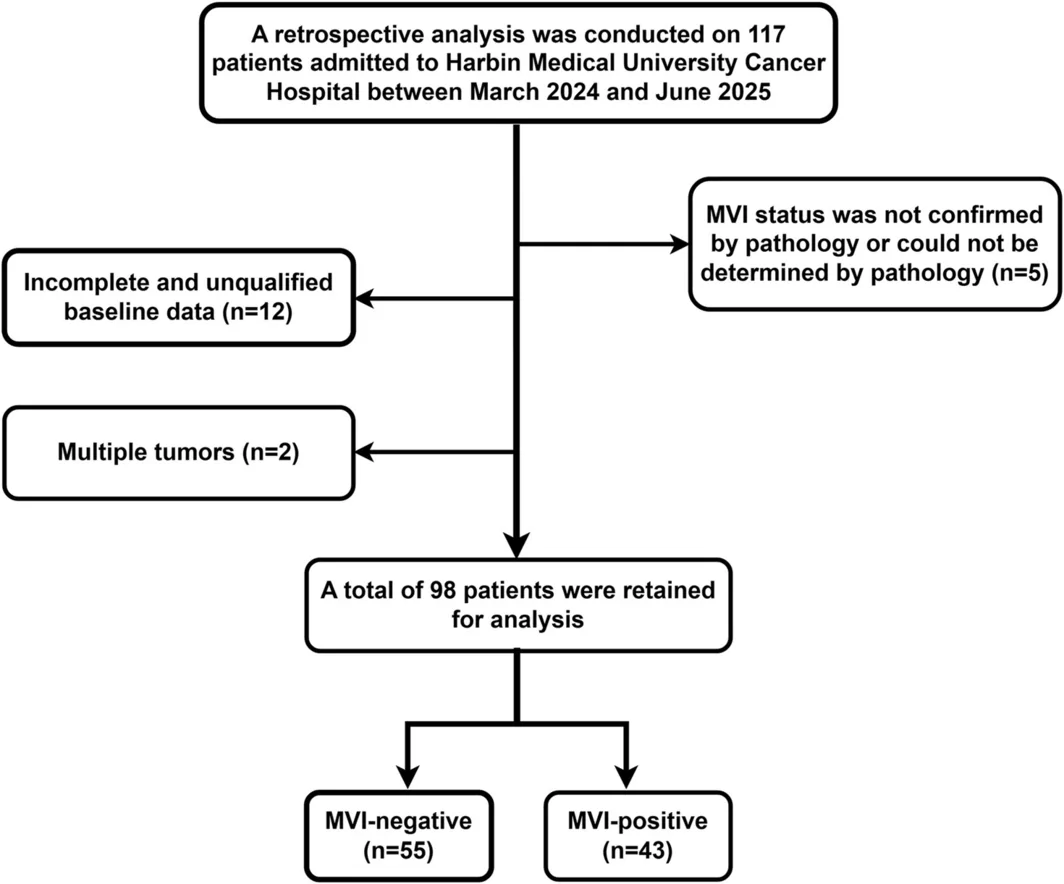
Flow chart of case inclusion and exclusion.

**TABLE 1:** General clinical data of patients in the two groups.

Variable	MVI‐negative group (*n* = 55)	MVI‐positive group (*n* = 43)	*p*
Age	59 (55, 66)	59 (51, 66)	0.600
Gender			0.795
Male	45 (80.36%)	36 (83.72%)	
Female	11 (19.64%)	7 (16.28%)	
Etiology of HCC			0.676
HBV	45 (81.82%)	38 (88.37%)	
HCV	3 (5.45%)	1 (2.33%)	
Other	7 (12.73%)	4 (9.30%)	
Antiviral therapy	42 (76.36%)	30 (69.77%)	0.496
Liver cirrhosis	35 (63.64%)	31 (72.09%)	0.396
AFP (ng/mL)			0.025
≥ 400	7 (12.73%)	14 (32.56%)	
< 400	48 (87.27%)	29 (67.44%)	
PIVKAII (mAu/mL)	125 (48.5, 357)	324 (62, 1189)	0.002
Albumin (g/L)	40.3 (37.8, 44)	40.2 (38.0, 41.4)	0.205
Total Bilirubin (g/L)	13.9 (10.3, 19.9)	16.0 (11.7, 20.6)	0.338
AST (U/L)	26 (20, 44)	33 (25, 57)	0.008
ALT (U/L)	21 (17, 37)	29 (21, 42)	0.067

Abbreviations: AFP, alpha‐fetoprotein; ALT, alanine aminotransferase; AST, aspartate aminotransferase; HBV, hepatitis B virus; MVI, microvascular invasion; PIVKAII, protein‐induced by vitamin K absence or antagonist‐II.

**FIGURE 2:**
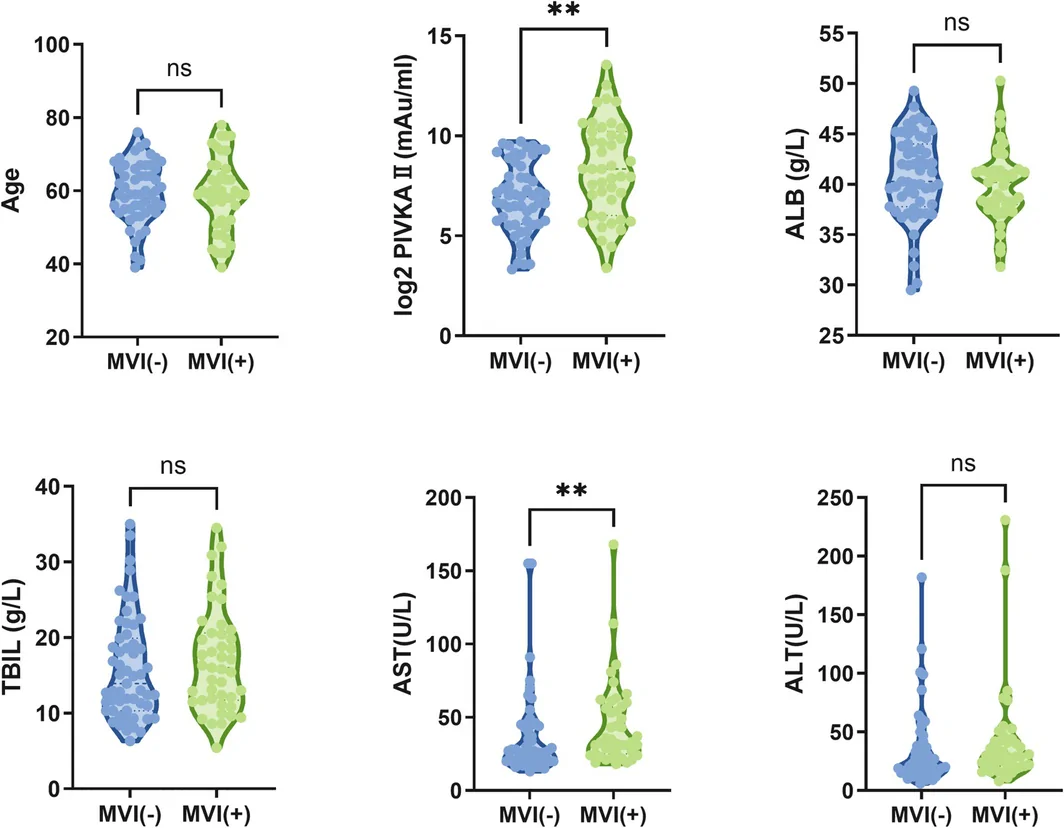
Baseline laboratory characteristics of the two patient groups. ALB, albumin; ALT, alanine aminotransferase; AST, aspartate aminotransferase; Log_2_ PIVKA‐II, base‐2 logarithm of protein induced by vitamin K absence or antagonist‐II; TBIL, total bilirubin. Statistical notations: ns, not significant (*p* > 0.05); **p < 0.01.

**FIGURE 3:**
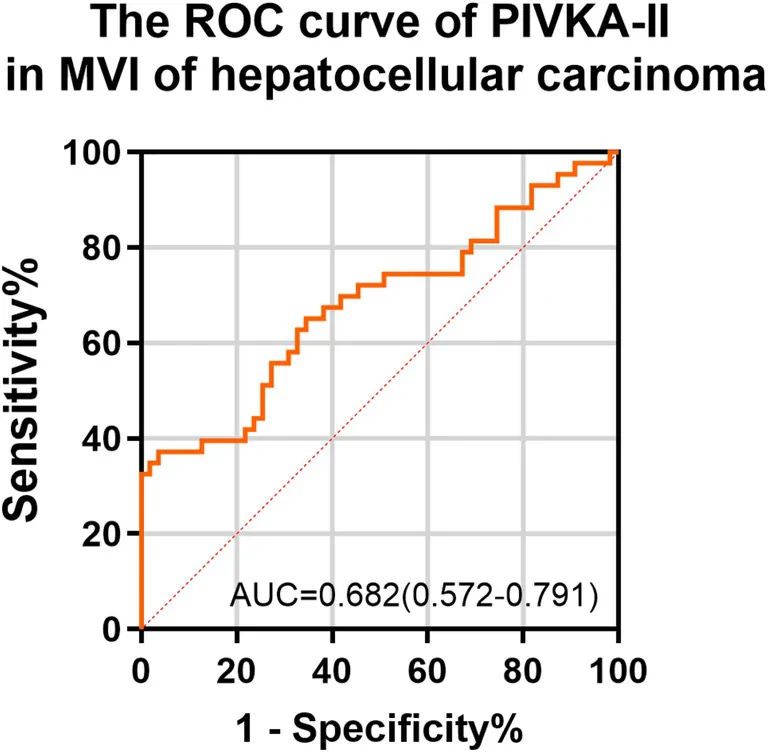
ROC curve of PIVKA‐II for predicting MVI in HCC patients. AUC, area under the curve; HCC, hepatocellular carcinoma; MVI, microvascular invasion; PIVKA‐II, protein induced by vitamin K absence or antagonist‐II; ROC, receiver operating characteristic.

### 3.2 Comparison of MRI Imaging Features Between MVI‐Positive and MVI‐Negative Groups

Analysis of MRI imaging features between the MVI‐negative group (55 cases) and the MVI‐positive group (43 cases) revealed no statistically significant differences in tumor capsule integrity (*p* = 0.509), T1WI hypointensity (*p* > 0.999), T2WI hyperintensity (*p* > 0.999), DWI/T2WI mismatch (present in 23.64% vs. 25.58%; *p* > 0.999), margin morphology (regular in 56.36% vs. 41.86%; *p* = 0.222), or intratumoral mosaic architecture (present in 61.82% vs. 76.74%; *p* = 0.131). Peritumoral abnormal enhancement demonstrated borderline significance (*p* = 0.050). Statistically significant differences were observed in tumor diameter (*p* = 0.001), peritumoral abnormal enhancement (*p* = 0.009), intratumoral arteries (present in 9.09% vs. 30.23%; *p* = 0.009), and mean ADC value of the tumor (1.15 × 10^−3^ mm^2^/s in the MVI‐negative group vs. 0.979 × 10^−3^ mm^2^/s in the MVI‐positive group; *p* = 0.014). The MVI‐positive group exhibited a higher prevalence of peritumoral abnormal enhancement and intratumoral arteries, as well as a lower mean ADC value. Detailed results are presented in Table 2.

**TABLE 2:** MRI imaging features of patients in the two groups.

Parameter	MVI‐negative group (*n* = 55)	MVI‐positive group (*n* = 43)	*p*
Tumor diameter (mm)	40 (30, 50)	47 (39, 85)	0.001
Tumor capsule			0.509
Intact	15	15	
Incomplete	40	28	
Tumor T1WI hypointensity	44	34	> 0.999
Tumor T2WI hyperintensity	52	39	> 0.999
Tumor DWI/T2WI mismatch			> 0.999
Present	13 (23.64%)	11 (25.58%)	
Absent	42 (76.36%)	32 (74.42%)	
Marginal morphology			0.222
Regular	31 (56.36%)	18 (41.86%)	
Irregular	24 (43.64%)	25 (58.14%)	
Peritumoral abnormal enhancement			0.050
Present	8 (14.55%)	14 (32.56%)	
Absent	47 (85.45%)	29 (67.44%)	
Intratumoral artery			< 0.001
Present	5 (9.09%)	32 (74.42%)	
Absent	50 (90.91%)	11 (25.58%)	
Intratumoral mosaic pattern			0.131
Present	34 (61.82%)	33 (76.74%)	
Absent	21 (38.18%)	10 (23.26%)	
Mean tumor ADC value (10^−3^ mm^2^/s)	1.15 (0.984, 1.266)	0.979 (0.880, 1.149)	0.014

Abbreviations: ADC, apparent diffusion coefficient; DWI, diffusion‐weighted imaging; MVI, microvascular invasion; T1WI, T1‐weighted imaging; T2WI, T2‐weighted imaging.

### 3.3 Multivariate Logistic Regression Analysis of Risk Factors for MVI in HCC Patients

Serum and MRI imaging features with *p* < 0.05 in univariate analysis were included as candidate variables in multivariate logistic regression. The presence of MVI (absent = 0, present = 1) was used as the dependent variable, with age (continuous) and gender (female = 0, male = 1) as adjustment variables. The independent variables included serum PIVKA‐II (< 741.5 mAU/mL = 0, ≥ 741.5 mAU/mL = 1), AFP (< 400 ng/mL = 0, ≥ 400 ng/mL = 1), AST (continuous), tumor diameter (continuous), peritumoral abnormal enhancement (absent = 1, present = 0), intratumoral arteries (absent = 1, present = 0), and mean tumor ADC value (continuous). Stepwise forward multivariate logistic regression was performed with a significance level of *p* < 0.05. The results showed that elevated PIVKA‐II (odds ratio [OR] = 22.98), presence of intratumoral arteries (OR = 38.01), and mean tumor ADC value (OR = 0.03) were independent influencing factors for MVI in HCC (*p* < 0.05). Elevated serum PIVKA‐II and the presence of intratumoral arteries significantly increased the risk of MVI, whereas a higher mean tumor ADC value was associated with reduced risk. Collinearity diagnostics indicated no multicollinearity among these independent factors (variance inflation factor [VIF] < 5). Detailed results are presented in Table 3.

**TABLE 3:** Univariate and multivariate logistic regression analyses of risk factors for microvascular invasion in patients with HCC.

Variables	Univariate analysis						Multivariate analysis				
	*β*	S.E	*Z*	*p*	OR (95% CI)		*β*	S.E	*Z*	*p*	OR (95% CI)
Gender											
Female					1.00 (Reference)						
Male	−0.48	0.62	−0.78	0.435	0.62 (0.18 ~ 2.08)						
Age (years)	0.03	0.03	1.08	0.281	1.03 (0.98 ~ 1.09)						
AST (U/L)	0.01	0.01	1.27	0.203	1.01 (0.99 ~ 1.03)						
ALT (U/L)	0.01	0.01	0.69	0.487	1.01 (0.99 ~ 1.02)						
AFP (ng/mL)											
< 400					1.00 (Reference)						
≥ 400	1.45	0.66	2.21	**0.027**	4.25 (1.18 ~ 15.35)						
Serum PIVKA‐II (mAU/mL)											
< 741.5					1.00 (Reference)						1.00 (Reference)
≥ 741.5	3.48	1.08	3.23	**0.001**	32.37 (3.92 ~ 267.52)		3.13	1.18	2.66	**0.008**	22.98 (2.28 ~ 231.21)
Peritumoral abnormal enhancement											
No					1.00 (Reference)						
Yes	0.83	0.60	1.38	0.166	2.29 (0.71 ~ 7.37)						
Intratumoral arteries											
No					1.00 (Reference)						1.00 (Reference)
Yes	3.30	0.72	4.58	**< 0.001**	27.22 (6.62 ~ 111.97)		3.64	0.92	3.96	**< 0.001**	38.01 (6.28 ~ 230.08)
Tumor diameter (mm)	0.04	0.01	2.99	**0.003**	1.04 (1.01 ~ 1.07)						
ADC value (×10^−3^ mm^2^/s)	−2.79	1.16	−2.40	**0.016**	0.06 (0.01 ~ 0.60)		−3.64	1.55	−2.35	**0.019**	0.03 (0.00 ~ 0.55)

*Note:* Variables with *p* < 0.05 in the univariate analysis were included in the multivariate analysis. In the multivariate analysis, variables with *p* < 0.05 were considered independent risk factors. Bolded *p*‐values indicate statistical significance (*p* < 0.05). For continuous variables (Age, AST, ALT, Tumor Diameter, ADC Value), the odds ratio represents the increased risk for each one‐unit increase.

Abbreviations: *β*, regression coefficient; ADC: apparent diffusion coefficient; AFP, alpha‐fetoprotein; ALT, alanine aminotransferase; AST, aspartate aminotransferase; CI: confidence interval; OR: odds ratio, *p*, *p* value; PIVKA‐II, protein‐induced by vitamin K absence or antagonist‐II; S.E., standard error; *Z*, *Z*‐statistic.

### 3.4 A Diagnostic Model for MVI in HCC Based on PIVKA‐II Combined With MRI Radiomic Features

A nomogram for predicting MVI in HCC was developed using PIVKA‐II, intratumoral arteries, and mean tumor ADC value (Figure 4A). The total score for each patient was obtained by summing the points assigned to each variable. Projecting the total score vertically to the risk axis indicated the individual probability of MVI. ROC curve analysis demonstrated that the nomogram model achieved an AUC of 0.81 (95% CI: 0.62–0.90) for predicting MVI, which was significantly higher than that of PIVKA‐II alone (Figure 4B). The Hosmer–Lemeshow goodness‐of‐fit test suggested no significant deviation from perfect fit (*χ* ^*2*^ = 5.5153, *p* = 0.7013 > 0.05). Furthermore, a calibration curve was constructed based on 1000 bootstrap resamples, demonstrating strong agreement between the bias‐corrected curve and the actual curve in both the training and validation sets, both of which closely approximated the ideal curve (Figure 4C). DCA revealed that the nomogram yielded a higher net benefit than the treat‐all and treat‐none strategies across a wide range of threshold probabilities. The model showed clinical utility for threshold probabilities between 0.18 and 1.00 (Figure 4D). [Correction added on 25 March 2026, after first online publication: In the first sentence “A nomogram for predicting MVI.....” the phrase “tumor diameter” has been deleted in this version.]

**FIGURE 4:**
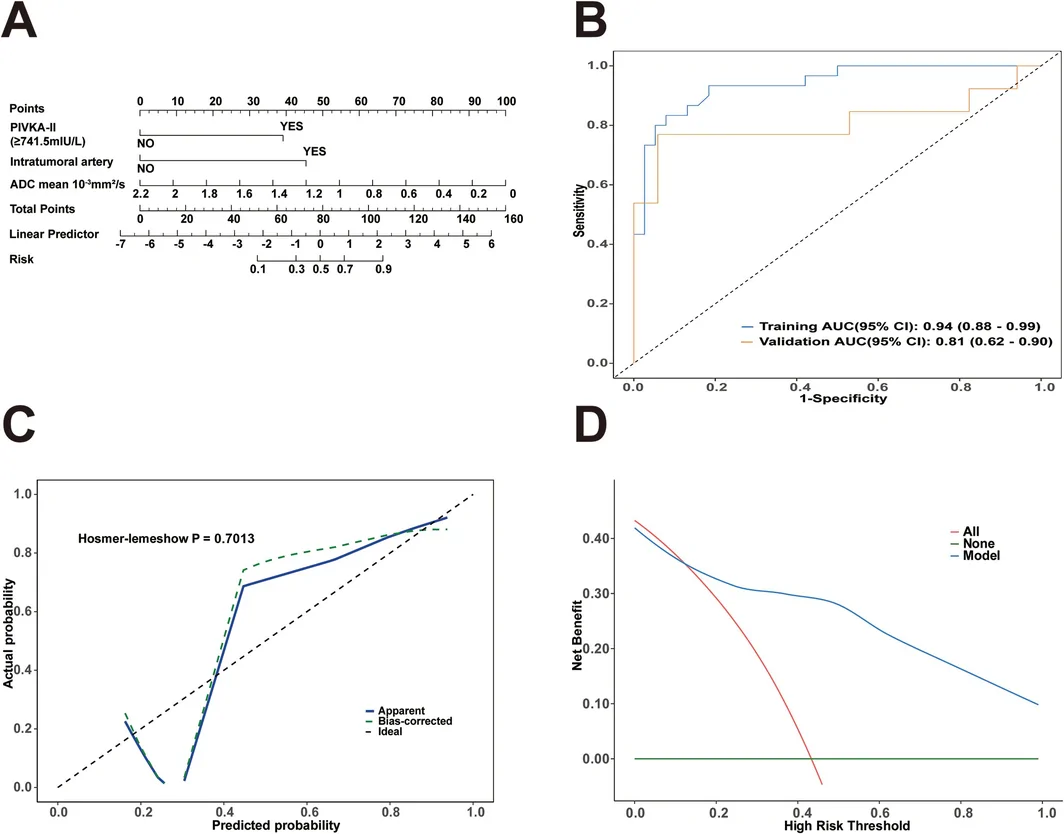
Diagnostic model for MVI in HCC patients constructed by combining PIVKA‐II with MRI radiomic features. (A) Nomogram of the model; (B) ROC curve; (C) Hosmer–Lemeshow calibration curve of the validation set; (D) decision curve analysis of the validation set. HCC, hepatocellular carcinoma; MRI, magnetic resonance imaging; MVI, microvascular invasion; PIVKA‐II, protein induced by vitamin K absence or antagonist‐II; ROC, receiver operating characteristic. [Correction added on 25 March 2026, after first online publication: Figure 4 has been replaced in this version.]

## 4 Discussion

Among patients with primary liver cancer, HCC accounts for more than 90% of cases. It is characterized by rapid progression, high metastatic potential, and poor prognosis. Early symptoms are often insidious, and most patients are diagnosed at an intermediate or advanced stage [25]. Radical resection remains the preferred treatment for early‐stage HCC patients with preserved liver function and has been shown to significantly prolong survival [26]. MVI is an independent risk factor for intrahepatic metastasis or recurrence after surgery in HCC patients [27]. MVI foci are often scattered in distribution; approximately 83.3% of MVI foci are located within 1 cm of the tumor, while about 8.4% are found in liver parenchyma more than 2 cm away from the tumor margin. However, not all patients can provide sufficient tumor samples for MVI assessment, especially those who undergo only biopsy. This limitation affects the accuracy of histopathological MVI evaluation and restricts its utility as a prognostic factor in clinical decision‐making [16]. Therefore, accurate preoperative prediction of MVI is essential for optimizing treatment strategies.

PIVKA‐II, also referred to as des‐gamma‐carboxy prothrombin (DCP), is an abnormal protein specifically produced in HCC and has been proposed as a potential tool for predicting vascular invasion, metastasis, and recurrence [28]. Previous studies have reported that a preoperative serum PIVKA‐II level greater than 40 mAU/mL can independently predict MVI risk in patients with solitary HCC tumors smaller than 3 cm [29]. Other studies have suggested optimal cutoff values of PIVKA‐II ≥ 90 mAU/mL [30] or PIVKA‐II > 200 mAU/mL [31] for MVI prediction. In this study, the optimal cutoff value of PIVKA‐II for MVI diagnosis was determined to be 741.5 mAU/mL, with a specificity of 96.36%, supporting its value in identifying highly aggressive tumors. However, its sensitivity of 37.21% indicates that using PIVKA‐II alone may lead to the underdiagnosis of patients with low expression, highlighting the need for its combination with other indicators. Although PIVKA‐II shows certain predictive value for MVI, there is no unified standard for its cutoff values, and the underlying mechanisms linking PIVKA‐II to MVI remain unclear. Fujikawa et al. [32] suggested that PIVKA‐II may enhance the proliferation and migration of vascular endothelial cells, thereby promoting tumor metastasis.

Current clinical diagnosis of liver cancer still relies heavily on imaging methods. Among various imaging modalities, MRI is the standard technique for diagnosing HCC according to international guidelines. Compared to other methods, MRI offers advantages such as the absence of ionizing radiation, high safety, excellent soft‐tissue contrast, superior tissue penetration, and high spatial resolution, making it a superior diagnostic tool for liver cancer [33]. MRI features provide additional predictive information from both morphological and functional perspectives. Some studies have found that the presence of intratumoral arteries increases the risk of MVI by 2.5 times [34]. Yang et al. [35] developed an intratumoral and peritumoral radiomics model and confirmed the correlation between intratumoral arteries and MVI. In this study, the presence of intratumoral arteries (30.23% in the MVI‐positive group vs. 9.09% in the MVI‐negative group) reflects the formation of abnormal vascular networks within the tumor. These vessels often lack structural integrity, providing direct pathways for tumor cells to enter the circulation. Previous studies have shown an inverse correlation between tumor ADC values and tumor cellular density—higher cellular density corresponds to lower ADC values [36]. Additionally, ADC value has been identified as a high‐risk factor for predicting MVI in HCC [37], indicating that HCC with MVI is more aggressive than well‐differentiated HCC without MVI. In this study, the lower mean tumor ADC value in the MVI‐positive group (0.979 × 10^−3^ mm^2^/s) is consistent with the established notion that low ADC values indicate high invasiveness. Tumor diameter, particularly the long‐axis diameter, is closely associated with MVI. It serves as an important indicator of tumor burden and is closely related to tumor cell proliferation, angiogenesis, and invasive potential. A larger tumor diameter is associated with richer neovascularization and a higher probability of vascular invasion and MVI. The important role of the tumor long‐axis diameter in predicting MVI has been confirmed by multiple studies [38, 39]. In this study, univariate analysis showed a statistically significant difference in tumor long‐axis diameter between MVI‐positive and MVI‐negative patients. However, in the subsequent stepwise backward logistic regression analysis, this indicator was not retained in the final model. A potential reason is that with a small sample size, the stability of the multifactorial model fitting may be suboptimal, leading to the preferential retention of variables showing a stronger association with MVI.

Compared to current single‐dimensional MVI prediction studies, the combined model in this study demonstrates significant advantages. In the field of serum biomarkers, most research has focused on AFP; however, AFP exhibits a positivity rate of only 60%–70% in early‐stage HCC and is influenced by benign liver diseases, limiting its specificity [40]. This study selected PIVKA‐II, which shows higher specificity for HCC, particularly in early‐stage small HCCs. When combined with MRI features, the model achieved an AUC of 0.81, representing an improvement of approximately 18.7% compared to using PIVKA‐II alone (AUC of 0.682), validating the necessity of multidimensional integration. Regarding imaging studies, previous research has predominantly emphasized morphological features such as tumor diameter and capsule integrity. This study, however, found that only tumor diameter, peritumoral abnormal enhancement, intratumoral arteries, and ADC value showed statistically significant differences, while capsule integrity and T1WI/T2WI signal characteristics showed no significant association. This may be attributed to the inclusion of patients classified as Child‐Pugh A and BCLC stage A, in whom early‐stage tumors often have intact capsules, thereby diminishing their predictive value. In contrast, functional indicators such as intratumoral arteries and ADC values better reflect tumor biological behavior, suggesting that functional features may have superior predictive value over morphological characteristics in early HCC. Model evaluation using the Hosmer–Lemeshow goodness‐of‐fit test (*p* = 0.699) indicated strong agreement between predicted probabilities and actual MVI incidence. DCA further confirmed that the model significantly improves the net treatment benefit within the medium‐to‐high risk threshold range (20%–60%), thereby helping to avoid both overtreatment and undertreatment. Additionally, the study strictly limited inclusion to Child‐Pugh A and BCLC stage A patients with solitary tumors—a population primarily considered for surgical intervention—making the results highly relevant for direct clinical application in early HCC management and providing clear guidance for surgical planning and postoperative adjuvant treatment decisions. [Correction added on 25 March 2026, after first online publication: In the sentence “When combined with MRI features, the mode …”, the values have been changed from “0.85” to “0.81” and from “24.6%” to “18.7%” in this version.]

Despite these meaningful results, several limitations should be addressed in future studies. First, the relatively small sample size is a key limitation of this study. The inclusion of 98 patients (43 MVI‐positive) may lead to suboptimal statistical power for a model containing multiple predictor variables, potentially affecting model stability, as possibly reflected in the wide confidence intervals of some ORs (e.g., 95% CI for intratumoral arteries: 5.41–249.26). This also raises a potential risk of overfitting. Furthermore, the single‐center retrospective design with a relatively short recruitment period (2024–2025) may have further limited sample accrual. The lack of external validation could also affect the generalizability of the model. Second, there is a potential risk of selection bias. By including only Child‐Pugh A, BCLC Stage A patients with solitary tumors, the study ensured cohort homogeneity but may have limited the representativeness of the sample. The model's applicability to patients with Child‐Pugh B/C cirrhosis, multifocal tumors, or those receiving non‐surgical treatments remains unclear and requires further validation. Finally, dichotomizing the continuous biomarker PIVKA‐II, while having some rationale, may lead to loss of information, potentially reducing predictive accuracy and exaggerating effect sizes—a methodological limitation worth noting. Future studies could address these limitations by: extending the recruitment period to increase the sample size (particularly MVI‐positive cases) for improved reliability and statistical power; conducting multicenter prospective studies including patients with varying liver function grades, tumor stages, and treatments to reduce selection bias; performing external validation to enhance generalizability; and comparing model performance with PIVKA‐II treated as a continuous versus dichotomous variable to inform optimal modeling strategies.

This study developed a preliminary prediction model with potential clinical applicability, offering a reference for preoperative MVI risk assessment in HCC. For patients identified by the model as high‐risk for MVI, extended hepatectomy or anatomical resection could be prioritized, accompanied by intensified postoperative monitoring. For low‐risk patients, local resection or laparoscopic minimally invasive surgery might be considered to reduce trauma. From a medical resource optimization perspective, the model could facilitate “precise monitoring and stratified management” through preoperative screening, thereby supporting rational resource allocation. It should be emphasized that, given the current limited sample size (*n* = 98), the model's stability and generalizability require further validation. Thus, the clinical applications suggested above are preliminary considerations based on available data and should not directly guide clinical decision‐making. The primary value of this study lies in validating the feasibility of predicting MVI by combining PIVKA‐II with MRI features, and in identifying key imaging markers such as intratumoral arteries and tumor ADC values. This provides an important reference for subsequent large‐sample, multicenter studies. Following future external validation, sample expansion, and indicator optimization, this model is expected to be further translated into clinical practice and play a greater role in the precise diagnosis and treatment of HCC.

## Ethics Statement

The present study was approved by the Ethics Committee of Harbin Medical University Cancer Hospital and written informed consent was provided by all patients prior to the study start. All procedures were performed in accordance with the ethical standards of the Institutional Review Board and The Declaration of Helsinki, and its later amendments or comparable ethical standards.

## Conflicts of Interest

The authors declare no conflicts of interest.

## Data Availability

The datasets used and/or analyzed during the present study are available from the corresponding author on reasonable request.
